# HIF1*α*-Induced Glycolysis in Macrophage Is Essential for the Protective Effect of Ouabain during Endotoxemia

**DOI:** 10.1155/2019/7136585

**Published:** 2019-04-28

**Authors:** Chao Shao, Shengwei Lin, Sudan Liu, Peipei Jin, Wenbin Lu, Na Li, Yan Zhang, Lulong Bo, Jinjun Bian

**Affiliations:** ^1^Faculty of Anesthesiology, Changhai Hospital, Naval Medical University, Shanghai 200433, China; ^2^Department of Anesthesiology, Urumqi General Hospital of Lanzhou Military Command, Urumqi 830000, China; ^3^Department of Anesthesiology, The North Hospital of Ruijin Hospital Affiliated to the Medical College of Shanghai Jiaotong University, Shanghai 201821, China

## Abstract

Ouabain, a steroid binding to the Na^+^/K^+^-ATPase, has several pharmacological effects. In addition to the recognized effects of blood pressure, there is more convincing evidence suggesting that ouabain is involved in immunologic functions and inflammation. Hypoxia-inducible factor 1*α* (HIF-1*α*) is a metabolic regulator which plays a considerable role in immune responses. Previous studies had shown that HIF-1*α*-induced glycolysis results in functional reshaping in macrophages. In this study, we investigated the role of glycolytic pathway activation in the anti-inflammatory effect of ouabain. We found that ouabain is involved in anti-inflammatory effects both in vivo and in vitro. Additionally, ouabain can inhibit LPS-induced upregulation of GLUT1 and HK2 at the transcriptional level. GM-CSF pretreatment almost completely reversed the inhibitory effect of ouabain on LPS-induced release of proinflammatory cytokines. Alterations in glycolytic pathway activation were required for the anti-inflammatory effect of ouabain. Ouabain can significantly inhibit the upregulation of HIF-1*α* at the protein level. Our results also revealed that the overexpression of HIF-1*α* can reverse the anti-inflammatory effect of ouabain. Thus, we conclude that the HIF-1*α*-dependent glycolytic pathway is essential for the anti-inflammatory effect of ouabain.

## 1. Introduction

Sepsis is the leading cause of death in ICU-hospitalized patients worldwide. Sepsis is defined as life-threatening organ dysfunction caused by a dysregulated host response to infections since 2016 [1]. It affects more than 19 million people every year [2]. In the last decade, there has been progress in the management of sepsis. However, the mortality rate of sepsis is still quite high, around 25-30%.

Infection-induced abnormal metabolism involves and contributes to sepsis and septic shock. At the beginning, it appears a hyperinflammatory period, when a large number of immune cells are activated and recruited into various organs. This is followed by hypoinflammatory reactions characterized by worsening metabolism, which can further lead to death [3]. Research efforts to clarify the causes of septic shock have focused on hypoxia and interactions between glycolysis and oxidative phosphorylation of affected cells.

Ouabain, a steroid binding to Na^+^/K^+^-ATPase (NKA), is a hormone secreted by the adrenal gland and hypothalamus [4, 5]. Since ouabain acts as a cardiotonic steroid hormone in higher mammals [6], a great deal of research has discovered the physiological and pharmacological effects of ouabain. Ouabain exerts its biological functions through binding to Na^+^/K^+^-ATP receptors on target cells. In addition to its role in the cardiovascular system and blood pressure management [7], recent researches revealed that ouabain was also engaged in the regulation of immunologic functions [8, 9]. Inflammation is a closely coordinated immune process. Ouabain has been reported to regulate many inflammatory events, including vascular permeability, cell migration, and cytokine production [10, 11]. In addition, ouabain also interferes with neuroinflammation [12, 13]. However, ouabain has a dual proinflammatory and anti-inflammatory effect [14], which depends mainly on the functional status of target cells and the concentration of ouabain. Low concentrations of ouabain as a cytokine immunomodulator can be used for different clinical conditions. Our previous studies have shown that low dose of ouabain reverses sepsis-induced immune paralysis by increasing the levels of TNF-*α*, IFN-*γ*, and GM-CSF, then results in an improvement of the survival rate and negatively modulates the severity of lipopolysaccharide- (LPS-) induced acute lung injury (ALI) in mice [15, 16].

Macrophages play a critical role in maintaining homeostasis of the immune system. Previous investigations indicated that 2-deoxyglucose, which can inhibit hexokinase at the first step of glycolysis, was applied in various contexts and was shown to inhibit macrophage activation in vitro and to suppress inflammation in animal models [17]. All of these studies indicated that glycolysis is crucial for the function of immune cells. Enhanced glycolysis occurs in LPS-activated macrophages, dendritic cells [18, 19], and activated effector T cells [20]. Thus, the increased level of glycolysis might be considered as a symbol of metabolic change in most immune cells undergoing rapid activation. For example, in response to the stimulation of the pattern recognition receptor (PRR), cytokine receptors, or antigen receptors, enhanced glycolysis facilitates the immune cells to generate enough ATP and biosynthetic intermediates to fulfill their specific functions, including phagocytosis and production of inflammatory cytokines in macrophages [21].

Cell metabolism has become a key mechanism in regulating inflammatory responses [21]. LPS-induced glucose transport in macrophages is mainly mediated by glucose transporter 1 (GLUT1) [22]. In addition, hexokinase (HK) is a key enzyme for glycolysis, and 2-deoxy-D-glucose (2-DG) competitively inhibits HK activity, which in turn reduces the glycolysis rate and reduces LPS-induced release of proinflammatory cytokines [23]. There are strong shreds of evidence which proved that glycolysis produces lactate [24].

Hypoxia-inducible factor 1 (HIF-1) is a heterodimeric transcription complex consisting of two subunits, *i.e.*, HIF-1*α* and HIF-1*β* [25]. HIF1*α* proteins are targeted for degradation in an oxygen-dependent manner under normoxic conditions. Cellular HIF1 levels are controlled in part through prolyl hydroxylase domain- (PHD-) mediated proteolytic degradation. Recently, it has been demonstrated that bacteria or LPS can enhance HIF1 stability even under normoxic conditions [26, 27]. LPS was shown to increase succinate production in macrophages leading to hydroxylase inhibition and subsequent HIF stabilization, which led to an increased synthesis of IL-1*β*, a key proinflammatory factor [23]. In the innate immune system, HIF is critical for myeloid cells (granulocytes, monocytes, and macrophages) in bacterial elimination, aggregation, and invasion of immune cells under hypoxia [28].

Glucose metabolism is associated with the protective effect of ouabain, but the mechanism remained unclear. In this study, we demonstrate that HIF1*α* inhibits glycolysis in macrophages under the shielding effects of ouabain, leading to the inhibition of proinflammatory cytokine production both in vitro and in vivo.

## 2. Materials and Methods

### 2.1. Reagents and Animals

RPMI 1640 medium was obtained from Invitrogen (CA, USA). Ouabain (purity ≥ 95%) and LPS (O55:B5) were from Sigma (St. Louis, MO, USA). Dimethyloxalylglycine (DMOG) was obtained from Sigma. Male C57BL/6 mice were obtained from the Laboratory Animal Center of Second Military Medical University (Shanghai, China). Animals were randomly assigned to experimental groups without labeling procedures, and blinded studies were used to minimize group bias and subjective bias in assessing outcomes. Animals were placed in standard cages at 25°C, in a 12/12 light-dark cycle in a clean room, and were fed with food and water. All animals received medical treatment on the basis of Chinese legal requirements, and the experiment was approved by the Animal Experimental Commission of Second Military Medical University.

### 2.2. LPS-Induced Endotoxemia Model and Therapeutic Interventions with Ouabain

C57BL/6 mice were divided into four groups (*n* = 6); groupings were considered as control (PBS), ouabain (0.56 mg/kg), LPS (5 mg/kg), and LPS (5 mg/kg) with ouabain (0.56 mg/kg body weight of mice). LPS solution was prepared in warmed PBS (37°C) and was injected intraperitoneally (IP) at a dose of 5 mg/kg body weight (b.w.). Ouabain was injected intraperitoneally (IP) at a dose of 0.56 mg/kg b.w. 30 minutes before LPS injection. After 6 hours, mice were anesthetized with sevoflurane; then, blood was harvested by cardiac puncture, and a liver and a lung were harvested by being fixed in 10% formalin for hematoxylin and eosin (H&E) staining. The sections were examined and scored through the light microscopy by two independent pathologists. The scoring system was described in the previous study [29]. Generally, for lung inflammation, the scoring is as follows: 0, normal; 1, minimal inflammatory changes; 2, no obvious damage to the lung architecture; 3, thickening of the alveolar septa; 4, formation of nodules or areas of pneumonitis that distorted the normal architecture; and 5, total obliteration of the field. For liver necrosis, the scoring is as follows: 0, normal; 1, individual cell necrosis; 2, up to 30% lobular necrosis; 3, up to 60% lobular necrosis; and 4, more than 60% lobular necrosis. Blood was centrifuged at 5000 rpm for 10 minutes.

### 2.3. Cell Culture

Bone marrow-derived cells from C57BL/6 mice were cultured in RPMI 1640 media supplemented with 10% FBS and 1% penicillin/streptomycin and differentiated to macrophages (bone marrow-derived macrophages (BMDMs)) by recombinant murine GM-CSF (25 ng/ml; Miltenyi Biotech) for 7 days. Peritoneal macrophages (PMs) were isolated from the peritoneal cavity 3 days after the intraperitoneal injection of a 3% thioglycollate solution (Fluka, Sigma-Aldrich, St. Louis, MO, USA). PMs, the murine-derived macrophage cell line RAW 264.7 cells, were cultured using RPMI 1640 (Gibco, Life Technologies, Carlsbad, CA, USA) supplemented with 1% (*v*/*v*) penicillin/streptomycin (Sigma-Aldrich). All types of cells were maintained at 37°C in a humidified atmosphere of 5% CO_2_.

### 2.4. Cytokine Detection

The accumulation of TNF-*α* and IL-6 in the culture medium or mouse serum was measured per triplicate using commercial kits (eBioscience, San Diego, CA), following the indications of the supplier.

### 2.5. Quantitative Real-Time PCR

For qRT-PCR, 4 × 10^5^ PMs were seeded in 24-well dishes and incubated as described. One to three hours after infection, cells were washed with PBS at room temperature and lysed in 500 ml TRIzol (Invitrogen Life Technologies, Carlsbad, CA).

Total RNA was extracted using chloroform (100 ml). For qPCR, cDNA was generated from 1 mg RNA using SuperScript III (Invitrogen Life Technologies, Carlsbad, CA) and oligo (dT) primers. Selected genes were analyzed using Maxima SYBR Green-qPCR Master Mix (Thermo Scientific, Waltham, MA). Each sample was analyzed in triplicate on a CFX96 real-time PCR detection system (Bio-Rad). *C*_Q_ values were normalized to GAPDH-derived values, and relative changes in gene expression were estimated using the 2-*C*_Q_ method. Specific primers are with the following sequences: HIF-1*α*, 5-GAAACGACCACTGCTAAGGCA-3 (forward) and 5-GGCAGACAGCTTAAGGCTCCT-3 (reverse).

### 2.6. Overexpression of HIF1*α*

With overexpressed HIF-1*α*, RAW264.7 macrophages were used. Cells at 50% confluency were placed in a fresh culture medium containing serum 2 h before transfection. Then, pCMV-HIF-1*α* plasmid was transfected into the cells for 24 h following the manufacturer's protocol. Lipofectamine™ 2000 Transfection Reagent (Thermo Fisher, Cat#11668-027) was used for HIF-1*α* overexpression. In each experiment, the efficiency of gene overexpression was measured by Western blot analysis.

### 2.7. Western Blot Analysis

PMs were isolated as described. LPS (100 ng/ml) were added to PMs for 0-1 h. Cells harvested and extracted with precipitation assay buffer were probed using rabbit anti-mouse HIF (Novus Biologicals) at 1/1000.

### 2.8. Bioenergetic Study

Macrophage glycolysis function was assessed using the Seahorse XF Glycolysis Stress Test Kit with a Seahorse XF96 Analyzer (Agilent Technologies, USA) following the manufacturer's instructions. Macrophages were seeded at a density of 3 × 10^6^/well onto the 96-well microplate (Agilent Technologies) for 2 h prior to the assay. The glycolytic activities were assessed by measuring the extracellular acidification rate (ECAR). Glycolysis is the ECAR after the addition of glucose. Glycolytic capacity is the increase in ECAR after the injection of oligomycin following glucose. The glycolytic reserve is the difference between glycolytic capacity and glycolysis. Data were normalized with the cell number and expressed as mpH/min/10^3^ cells (ECAR).

### 2.9. Statistical Analysis

All data were analyzed and processed using statistical software such as SPSS 17.0 (SPSS Inc., Chicago, IL, USA) and Prism 5.0 (GraphPad Software, USA). Experimental data were given as the mean ± standard deviation. Two differences in the groups were compared and analyzed by Student's *t*-test. To analyze differences in multiple groups, one-way ANOVA was used and the *p* value < 0.05 was used to indicate a statistically significant difference.

## 3. Results

### 3.1. The Protective Effects of Ouabain in LPS-Induced Endotoxemia

**Figure 1:**
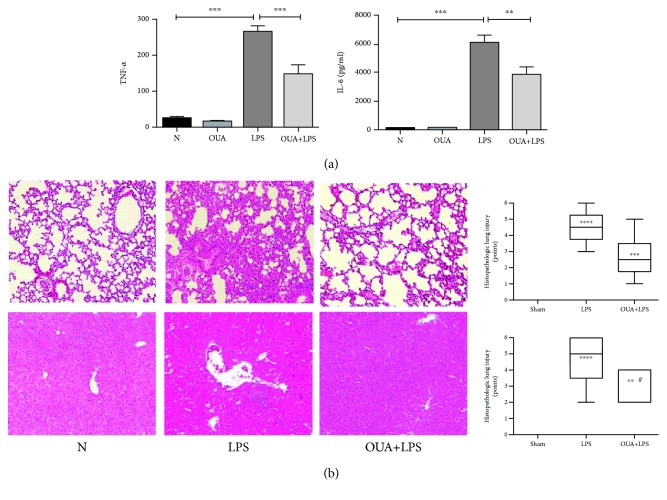
The effects of ouabain in LPS-induced endotoxemia and H&E staining for the liver and lung tissue sections of mice. Mice (*n* = 6) were pretreated with 0.56 mg/kg of ouabain (i.p.) or PBS. Half an hour after ouabain treatment, animals were administered with 5 mg/kg of LPS (i.p.). (a) The results indicated the level of proinflammatory cytokines in the serum of mice. (b) The results indicated the H&E staining for mouse liver and lung tissue sections and quantification of tissue damage. Results were expressed as mean ± SEM and analyzed by GraphPad Prism using ANOVA, where all groups were compared. ^∗∗∗^*P* < 0.001 and ^∗∗^*P* < 0.01.

To explore the role of ouabain in LPS-induced endotoxemia, we analyzed the production of inflammatory cytokines including TNF-*α* and IL-6 in C57BL/6 mice. LPS-induced endotoxemia model was established by intraperitoneal injection of LPS at a dose of 5 mg/kg. Thirty minutes before LPS injection, an equal volume of PBS or ouabain (0.56 mg/kg) was injected intraperitoneally (IP) to the mice. Our results suggest that LPS caused a significant increase in serum TNF-*α* and IL-6 levels in the endotoxemia model group, whereas pretreatment with ouabain significantly abolished this phenomenon (Figure 1(a)).

To verify the level of inflammation, we performed H&E staining of liver and lung tissue sections. We observed that in the control group, mice of which were pretreated with PBS then injected with LPS; the injury level both in the liver and the lung was significantly aggravated. Interestingly, the level of injury in the ouabain pretreated group was much more attenuated compared with the PBS-pretreated group (Figure 1(b)).

### 3.2. Effect of Ouabain on the Production of Proinflammatory Cytokines in Macrophages

**Figure 2:**
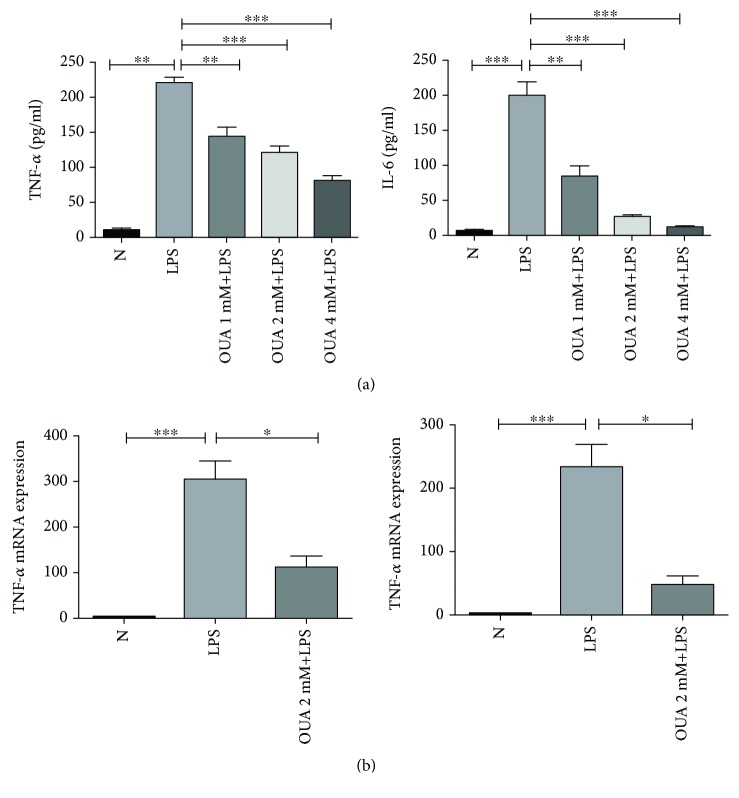
Effect of ouabain on the production of proinflammatory cytokines in macrophages. (a) ELISA experiments to illustrate the effect of ouabain on the production of proinflammatory cytokines in macrophage. (b) Detection of the effect of ouabain on macrophage mRNA of proinflammatory cytokines by qRT-PCR. Data were expressed as mean ± SEM and analyzed by GraphPad Prism using ANOVA with Tukey's posttest, where all groups were compared. ^∗∗∗^*P* < 0.001, ^∗∗^*P* < 0.01, and ^∗^*P* < 0.05.

Macrophages play a crucial role in the innate immune system. After being activated, macrophages release a large number of proinflammatory cytokines, including TNF-*α* and IL-6. In order to verify whether ouabain also has an anti-inflammatory effect in macrophages, we performed the next part of experiments. Macrophages were isolated and cultured in a 6/12/24-well plate. Cells were allowed to grow to confluence prior to ouabain or LPS administration, followed by PBS/ouabain pretreatment. Thirty minutes after ouabain treatment, cells were exposed to 100 ng/ml LPS. The supernatant was collected 6 h after LPS stimulation, and the expression levels of TNF-*α* and IL-6 were measured by ELISA. As expected, the levels of proinflammatory cytokine in the PBS-pretreated group were significantly increased, while the levels of proinflammatory cytokine in ouabain-pretreated group were lower compared with the PBS-pretreated group. These results indicate that ouabain could inhibit the production of proinflammatory cytokines in macrophages in a dose-dependent manner (Figure 2(a)). Additionally, the levels of TNF-*α* and IL-6 mRNA in peritoneal macrophages (PMs) were measured by qRT-PCR. As expected, TNF-*α* and IL-6 mRNA were significantly increased after LPS treatment, while this upregulation effect of LPS can be abolished by ouabain pretreatment (Figure 2(b)).

### 3.3. Effects of Ouabain on Glycolysis in Macrophages

**Figure 3:**
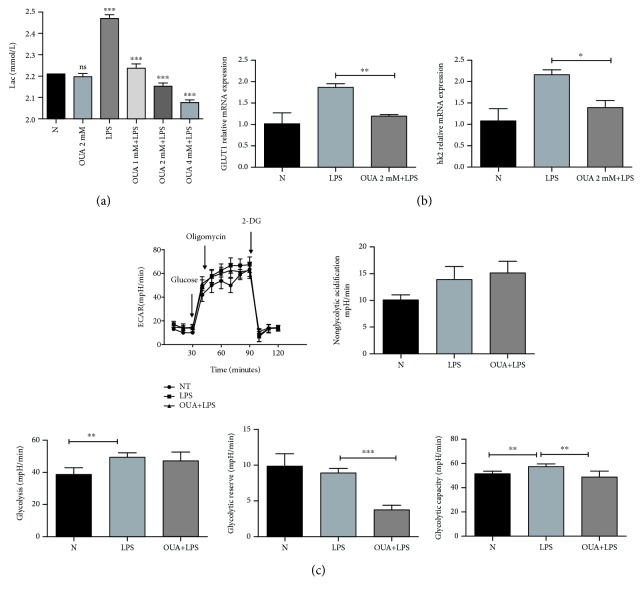
Effects of ouabain on glycolysis of macrophages. PMs from WT mice were stimulated with LPS (100 ng/ml) for 1–3 h, and the indicated mRNA expression was determined with qPCR. (a) The supernatant was collected, and the concentration of lactic acid was determined with spectrophotometry. (b) GLUT1 and HK2 mRNA expressions were determined with qPCR. (c) A representative graph output from XF96 showing ECAR response to glucose (10 mM), oligomycin (10 mM), and 2-DG (100 mM). *n* = 5. BMDMs were primed by 25 ng/ml GM-CSF for 24 h. BMDMs were seeded in a 96-well plate for 2 h, and glycolytic activities were examined using the XF Analyzer. (A) Nonglycolytic acidification. (B) Glycolysis. (C) Glycolytic reserve. (D) Glycolytic capacity. Mean ± SEM; ^∗^*P* < 0.05, ^∗∗^*P* < 0.01, and ^∗∗∗^*P* < 0.001, Student's *t*-test.

We checked the effect of ouabain on LPS-induced lactate production in supernatants of PMs. Compared with the PBS-pretreated group, lactic acid levels in the ouabain-pretreated group decreased significantly (Figure 3(a)). We further explored whether ouabain inhibits LPS-induced macrophage activation by inhibiting the expression of the glucose transporter and key enzymes in glycolysis. The results suggested that ouabain could inhibit LPS-induced upregulation of GLUT1 and HK2 at the mRNA level at 1 hour after LPS stimulation (Figure 3(b)).

The Seahorse XF glycolysis stress test reveals critical information about glycolysis pathways, such as lactic acid and ATP production, by measuring glycolysis, glycolytic capacity, and glycolysis reserves. Our results showed that LPS stimulation induced a significant increase in the extracellular acidification rate (ECAR) of bone marrow-derived macrophages (BMDMs), whereas in the case of ouabain pretreatment, ECAR showed a decreasing trend (Figure 3(c)). These results suggested that ouabain pretreatment could inhibit the enhanced glycolysis induced by LPS in BMDMs.

**Figure 4:**
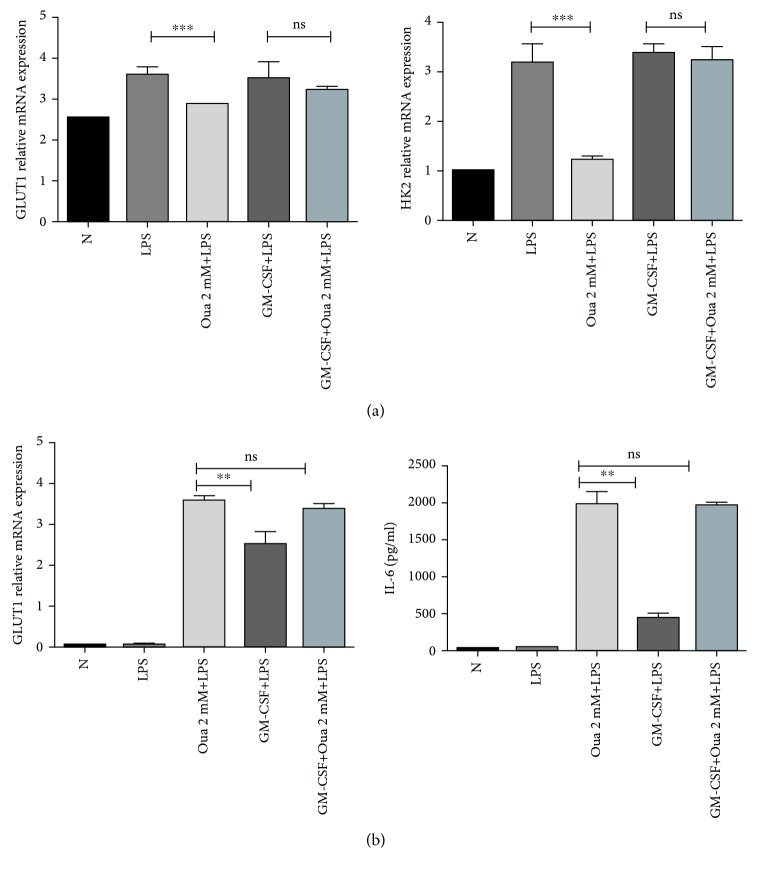
GM-CSF-mediated GLUT1 and HK2 mRNA expression and proinflammatory cytokine production. Cells were stimulated with LPS (100 ng/ml) for 6–8 h followed by GM-CSF (100 ng/ml) stimulation for 3 h. (a) GLUT1 and HK2 mRNA expressions were determined with qPCR (b) TNF-*α* and IL-6 were determined by ELISA. *n* = 4. ^∗∗^*P* < 0.005 and ^∗∗∗^*P* < 0.001, ANOVA.

Then, we used GM-CSF to enhance glycolysis to further verify whether glycolysis was necessary for the anti-inflammatory effect of ouabain. The results showed that GM-CSF pretreatment almost completely reversed the inhibitory effect of ouabain on LPS-induced release of proinflammatory cytokines (Figure 4(a)). As expected, GM-CSF pretreatment can almost completely blunt the inhibitory effect of ouabain on LPS-induced GLUT1 and HK2 upregulation at the mRNA level (Figure 4(b)).

### 3.4. HIF1*α* Is Required for Regulating the Anti-inflammatory Effect of Ouabain

**Figure 5:**
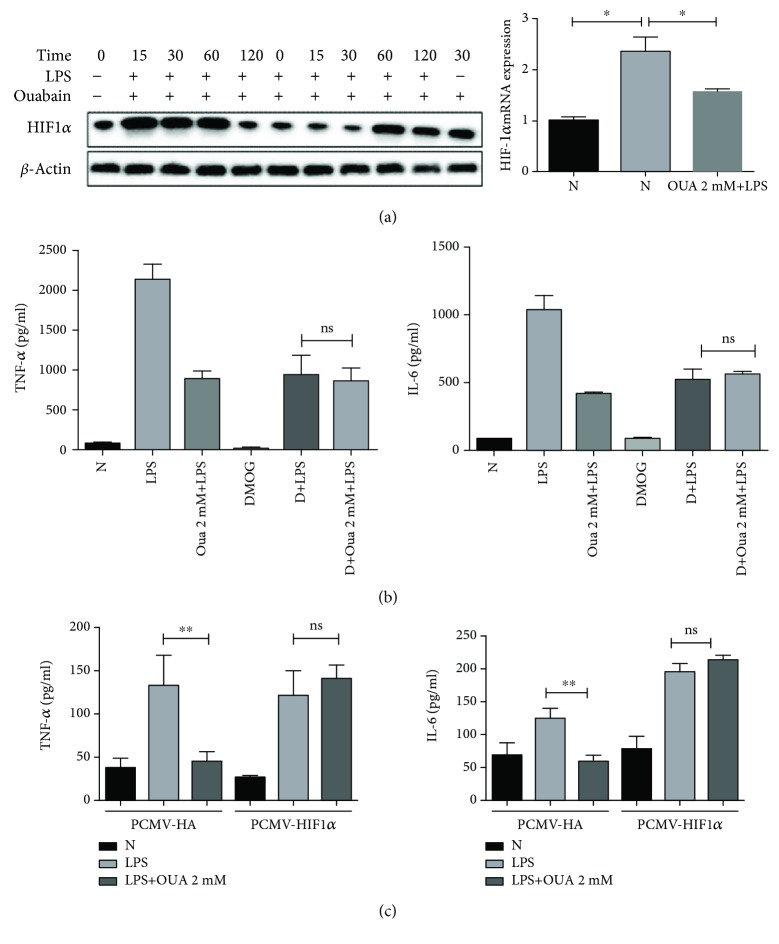
HIF1*α* is required for regulating the anti-inflammatory effect of ouabain. (a) PMs were examined for HIF1*α* protein levels (left) and gene transcripts (right) at the indicated time points using Western blot and real-time PCR. (b) Cells were stimulated with LPS (100 ng/ml) for 6–8 h followed by DMOG (0.5 mmol/l) stimulation for 3 h. TNF-*α* and IL-6 were determined by ELISA. (c) RAW264.7 cell lines transfected with plasmid-transfected HIF1*α* specific vector and blank vector. 24 h later, cells were treated with 2 mmol/l ouabain for 0.5 h and then stimulated with LPS for 6 h. Supernatants were assayed for TNF-*α* and IL-6 levels by ELISA. *n* = 4. ^∗∗^*P* < 0.005, ANOVA.

In previous experiments, we found that ouabain could inhibit the mRNA expression of GLUT1 and HK2. We further explored whether ouabain inhibits LPS-induced macrophage activation by inhibiting the expression of HIF-1*α*, the upstream regulator of glycolysis, through qRT-PCR and WB. Our results showed that HIF-1*α* mRNA expression was significantly reduced in the ouabain-pretreated group compared with the LPS-pretreated group (Figure 5(a)). Additionally, ouabain could significantly inhibit the upregulation trend of HIF-1*α* at the protein level. Dimethyloxalylglycine (DMOG), a competitive inhibitor of HIF-1*α* prolyl hydroxylase, stabilized the expression of HIF-1*α*. Our results showed that DMOG reversed the inhibitory effect of ouabain on LPS-induced release of proinflammatory cytokines in macrophages (Figure 5(b)).

The previous experiments confirmed that ouabain can inhibit the upregulation of HIF-1*α* both at the mRNA and protein levels. Therefore, we further observed whether the anti-inflammatory effect of ouabain could be reversed by overexpressing HIF1*α* in RAW264.7 cell. RAW264.7 cell was cultured and then transfected with HIF-1*α* plasmid and blank vector for 24 h; then, the cells were treated with 2 mmol/l ouabain for 0.5 h before exposing to 100 ng/ml LPS for 6 h. Cells were collected, and the supernatants were assayed for TNF-*α* and IL-6 levels by ELISA. The results revealed that the overexpression of HIF-1*α* by plasmid transfection also reversed the anti-inflammatory effect of ouabain (Figure 5(c)). In conclusion, it was confirmed that HIF-1*α* is necessary for ouabain to exert its anti-inflammatory action.

## 4. Discussions

Ouabain can regulate many inflammatory events, such as cell vascular permeability, migration, and cytokine production [10, 11]. Ouabain has a dual proinflammatory and anti-inflammatory effect [14, 15], which mainly depends on the functional status of cells and the concentrations of ouabain. Our study confirmed that ouabain can significantly reduce the release of proinflammatory cytokines induced by LPS and reduce the degree of liver and lung tissue injury in the model of LPS-induced endotoxemia mimicking sepsis.

During the past decades, numerous notable advances have been made in the understanding of endogenous ouabain, its receptor, and the downstream effects of activation of endogenous ouabain in the brain and periphery. Although many important questions remain to be investigated, compelling evidence indicates that endogenous ouabain is a significant entity in physiology and contributes to the pathogenesis of many common diseases [8]. At the same time, we also recognize that the content of endogenous ouabain in the body is so low that many methods of detection cannot accurately analyze the content. In vitro, we verified that the concentration of ouabain we used is not toxic to cells. In vivo, the dose of 0.56 mg/kg was chosen because using a different model this concentration was effective.

Direct effects of ouabain are originated due to the binding to its receptor, the Na^+^/K^+^-ATPase, on target cells. This interaction can promote Na^+^ transport blockade or even activation of signaling transduction pathways, *e.g.*, EGFR/Src-Ras-ERK pathway activation, independent of ion transport. A question that sometimes comes up is whether the inhibition of isolated ouabain-bound NKA molecules may mediate its downstream effects via local increases in sodium concentration. We find this is unlikely, taking into account that diffusion of ions in the cytoplasm is orders of magnitude faster than the slow turnover of isolated NKA. The NKA has a turnover on the millisecond scale, where the diffusion equilibration radius for cytoplasmic ions is on the micrometer scale [30].

Studies have shown that ouabain plays a significant role in glucose metabolism in the functional fate of immune cells. Naive or tolerant cells mainly rely on oxidative phosphorylation and *β*-oxidation as energy sources, whereas leukocytes transfer metabolism to aerobic glycolysis after acute phase stimulation (Warburg effect) [31]. The transition from oxidative phosphorylation to aerobic effects during the infection is an important host defense mechanism [32]. Infections and metabolic disorders are intertwined. For example, inhibiting metabolic pathways affects the immune response. Next, we want to explore the effects of glucose metabolism pathways on the anti-inflammatory effects of ouabain. Our results show that lactic acid, the final product of glycolysis, in the OUA+LPS group is lower than that of the LPS group. GLUT1, the glucose transporter, and HK2, the key enzyme for glycolysis, show the same trend as lactic acid. At the same time, ECAR as a credible standard for testing glycolysis also confirms this observation. Additionally, results on the oxygen consumption rate (OCR), regarding maximum respiration and spare respiratory capacity, showed no statistical difference between the OUA+LPS group and the LPS group (data not shown). It has been reported that GM-CSF enhances glycolysis in vivo and in vitro [33]. Therefore, we used GM-CSF to further verify whether glycolysis was necessary for the anti-inflammatory effect of ouabain. The results showed that GM-CSF pretreatment almost completely reversed the inhibitory effect of ouabain on LPS-induced release of proinflammatory cytokines.

The HIF pathway is one of the key transcriptional regulators in immunity and inflammation [34]. In addition, HIF-1*α* deletion mouse model has also shown the importance of HIF-1*α* for the function of the acute innate immune cell. HIF-1*α* conditional deletion in myeloid cells does not cause embryonic lethality in a mouse model nor does it has any defects in the development and differentiation of monocytes or neutrophils. However, these mice suffer from cell metabolism defects, which result in the inability of bone marrow cells to fully upregulate glycolysis in response to inflammatory stimulation [35]. In the absence of HIF-1*α*, macrophages exhibit a reduced glycolysis rate and dropped energy production [28]. Loss of HIF-1*α* in macrophages results in impaired phagocytic uptake and killing capacity in diverse bacterial infection models [36]. Mice with bone marrow cell-specific deletions of HIF-1*α* show a significant resistance to LPS-induced lethality, confirming the importance of HIF-1*α* in a classical innate immune model [37].

HIF-1*α* is a metabolic regulator that plays important roles in inflammation [38]. Additionally, HIF-1*α* impaired mitochondrial oxidation and promoted glycolysis metabolism in macrophages. Metabolic mechanisms in HIF-1*α*-deficient mice were reported to be accompanied with abolished glycolysis, decreased hepatic glucose output, and elevated gluconeogenesis [39]. Recent research confirmed that the HIF-1*α*-dependent glycolytic pathway is essential for the macrophage functional differentiation in protecting against bacterial and fungal infections [40]. We explored whether ouabain inhibits LPS-induced macrophage activation by inhibiting the expression of HIF1*α*, the upstream regulator of glycolysis, through qRT-PCR and WB. Our results showed that the HIF-1*α* mRNA expression and the protein level were significantly reduced in the ouabain group compared with the LPS group. Additionally, the overexpression of HIF-1*α* by plasmid transfection also reversed the anti-inflammatory effect of ouabain. In conclusion, it was confirmed that HIF-1*α* is necessary for ouabain to exert its anti-inflammatory effect.

Our study also has limitations. The results in vivo are relatively weak. We should next use HIF-1*α* gene knockout mice as well as overexpression of HIF-1*α* in vivo to further validate the mechanism of the anti-inflammatory effects of ouabain and use GM-CSF in vivo to enhance glycolysis to further explore the glycolysis pathway in the effect of ouabain. In animal experiments, we can then conduct HIF-1*α* interference experiments to further verify the mechanism to make the results more convincing. Cardiac glycosides have a number of pharmacological activities [9, 41]. Endogenous glycosides, including endogenous ouabain, are detectable in critically ill patients, especially in those with high risk of mortality [42]. Thus, cardiac glycosides are promising agents for sepsis therapy that deserve further investigation.

## 5. Conclusions

In the present study, we showed that ouabain can significantly inhibit the upregulation of HIF-1*α* at the protein level, revealing that the overexpression of HIF-1*α* can reverse the anti-inflammatory effect of ouabain during endotoxemia. HIF-1*α*-dependent glycolytic pathway is essential for the anti-inflammatory effect of ouabain during endotoxemia.

## Data Availability

The data including H&E staining of the liver and lung tissues, expression of TNF-alpha and IL-6, concentration of lactic acid, expression of GLUT1 and HK2, Western blot, expression of HIF1*α*, and extracellular acidification rates used to support the findings of this study are included within the article.
